# Effect of Ring Composition on the Statics and Dynamics of Block Copolyelectrolyte Catenanes

**DOI:** 10.1021/acs.macromol.5c00099

**Published:** 2025-04-18

**Authors:** Pietro Chiarantoni, Andrea Tagliabue, Massimo Mella, Cristian Micheletti

**Affiliations:** † Scuola Internazionale Superiore di Studi Avanzati - SISSA, via Bonomea 265, Trieste 34136, Italy; ‡ Institute for Computational Molecular Science, Temple University, 1925 N. 12th St., Philadelphia, Pennsylvania 19122, United States; § Dipartimento di Scienza e Alta Tecnologia, Università degli Studi dell’Insubria, Via Valleggio 11, Como 22100, Italy; ∥ Dipartimento di Fisica, Università di Genova, via Dodecaneso 33, Genoa 16146, Italy

## Abstract

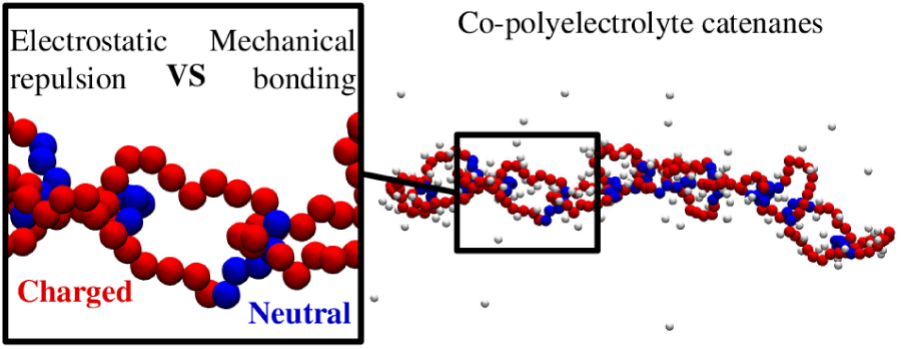

We use Langevin simulations to study the effect of ring composition on the structure and dynamics of model polycatenanes with copolyelectrolyte rings, each made of one charged and one neutral block. Key observables have a nonmonotonic dependence on ring composition, including the radius of gyration, mechanical bond length, orientational correlations, and rotational relaxation times. Microscopic analysis shows that these nonmonotonicities arise from the competition between electrostatic repulsion, pulling rings apart, and topological constraints, enforcing the proximity of neighboring rings. By locking charged-neutral interfaces at the mechanically bonded regions, this interplay can induce a strong chemical orientational order along the catenane while also hindering the local relaxation dynamics. Chemical orientation defects, manifesting as neutral–neutral interfaces, can emerge too and migrate along the catenane via coupled reorientations of neighboring rings. Our results clarify how ring composition and mechanical bonds can define the properties of topological materials across different scales.

## Introduction

Topological metamaterials, extended assemblies of mechanically interlocked molecules,^1−4^ were reportedly envisioned more than a century ago.^5^ However, it is only recently that breakthroughs in synthetic chemistry and combinatorial molecular design have enabled the high-yield production of supramolecular constructs with polymer-like connectivity through mechanical bonding. Examples range from linear catenanes^6,7^ and two-dimensional topological membranes^8,9^ to three-dimensional regular or irregular networks of interlocked molecules.^10−12^

Single-molecule probes, often complemented by modeling and simulations, are providing increasingly detailed insight into the fundamentally distinct properties of mechanically bonded systems and their covalently bonded counterparts. Differences have been reported across the main physical observables, such as metric scaling,^13−16^ intrinsic flexibility, writhe and curvature,^17−20^ relaxation dynamics,^21−23^ and the response to spatial confinement,^24−26^ molecular crowding^27^ or external forces.^6,28−33^ Besides being interesting *per se*, mechanically bonded structures are attracting interest for the possibility of harnessing topological constraints for tuning their physical properties in ways not available for conventional materials. A promising class of these tunable systems consists of interlocked block copolyelectrolytes (co-PEs), polymer chains with charged and neutral segments. These systems provide distinctive advantages for designability and external tunability.^34,35^ Charged blocks make the system’s size, shape, and dynamics responsive to external fields and to the concentration and valence of the ionic solution even in the presence of topological constraints.^36−43^ In addition, since topologically constrained co-PE strands primarily contact at their neutral regions,^34,35^ varying the neutral block length ought to allow for fine control over the geometry of mechanical bonding. Although these effects could provide considerable latitude for designing tunable topological materials, this potential has yet to be directly investigated. In fact, the notable properties of co-PE based materials^44−48^ have been mostly explored using the linear form of these molecules where, however, the effects of topological entanglements cannot be addressed.

Motivated by these considerations, here we examine the simplest type of topological materials comprising charged and neutral blocks: linear co-PE catenanes. Using Langevin molecular dynamics simulations, we study chains of interlocked ring polymers, each made of one charged and one neutral block, along with counterions that maintain the system’s overall charge neutrality. By systematically varying the relative size of these blocks, we analyze how this key design parameter influences the metric and dynamical properties of the catenane across various scales.

We show that several metric and dynamical observables have a nonmonotonic dependence on ring composition, including the radius of gyration, mechanical bond length, orientational correlations, and rotational relaxation times. Analysis of the catenane’s microscopic organization reveals that these nonmonotonicities arise from a competition between the intra- and inter-ring electrostatic repulsion and the mechanical bonding. This interplay favors the locking of charged-neutral interfaces at mechanically bonded regions, which can severely restrict the rings’ configurational space. This produces a strong chemical orientational order of the catenated rings while also hindering their rotational relaxation dynamics. Finally, our analysis reveals the presence of chemical orientation defects, manifesting as neutral–neutral interfaces at mechanically bonded regions. The defect dynamics involve concerted reorientations of neighboring rings, resulting in a cascade of defect hoppings. The cascade highlights the role of mechanical bonding in coupling structural reorganizations across various lengths and time scales.

## Methods

### Model and Simulation Setup

**Figure 1:**
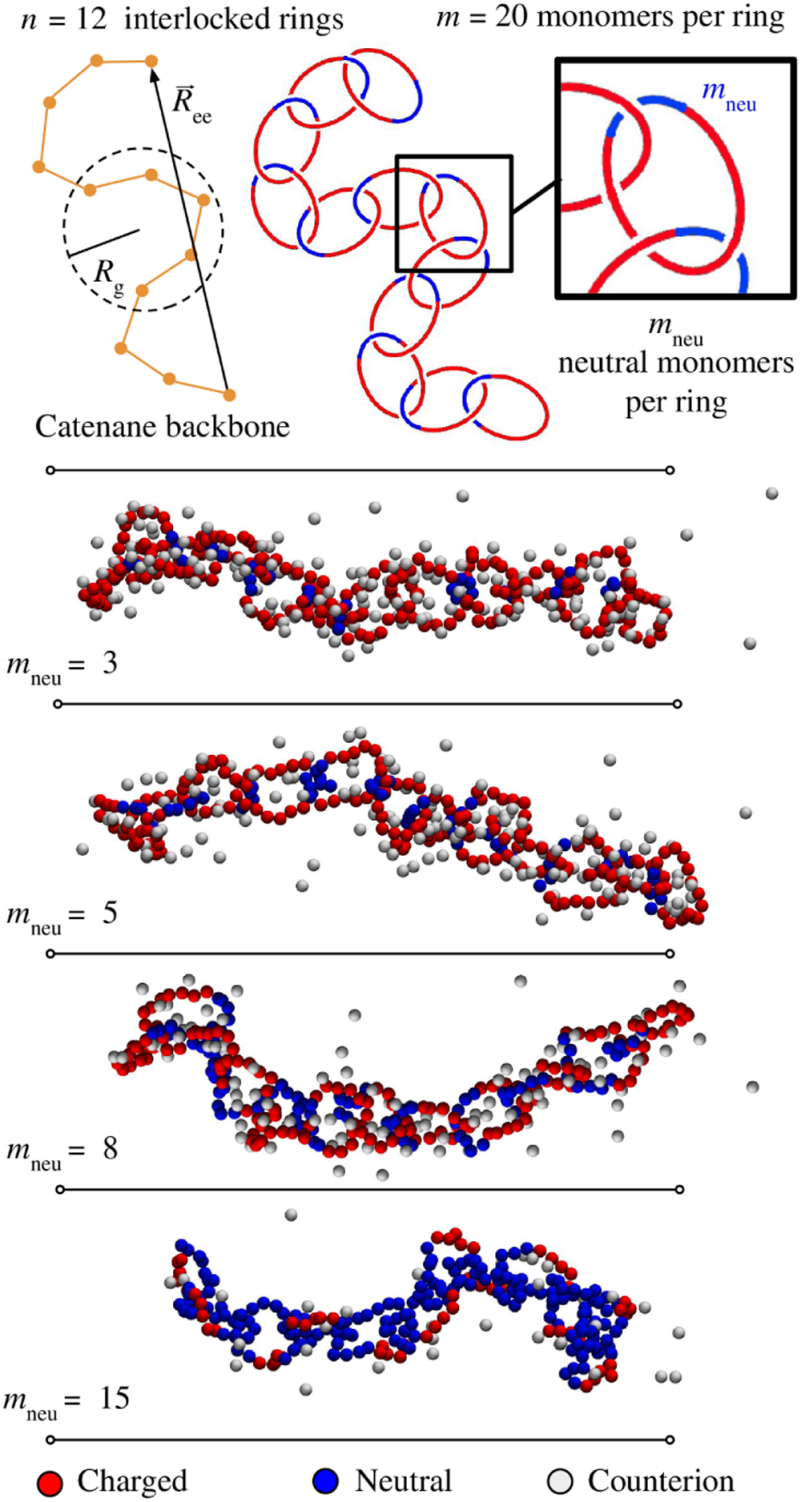
Typical configurations of the considered copolyelectrolyte linear catenanes at different ring compositions. The catenanes are made of *n* = 12 rings, each of *m* = 20 monomers, see sketch. The rings are diblock copolyelectrolytes, with one neutral block of *m*_neu_ monomers (blue) and a charged one of 20 – *m*_neu_ unit-charge monomers (red). Monovalent counterions (gray) ensure the overall charge neutrality of the system.

The simulated system consisted of a single linear polycatenane composed of *n* = 12 diblock copolyelectrolyte (co-PEs) rings, each made of *m* = 20 monomers, as illustrated in Figure 1, contained in a periodic cubic cell of side length *L*_box_. Each ring comprised two different blocks: a neutral segment of length *m*_neu_ and a charged segment of length *m*-*m*_neu_, and was modeled via a coarse-grained beads-and-springs representation. The overall charge neutrality of the system was maintained by the presence of *n*(*m* – *m*_neu_) monovalent counterions.

All particles had the same size, σ, and their excluded volume was modeled via the purely repulsive Weeks–Chandler–Anderson (WCA) interactions,^49^
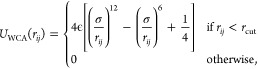
1where *r*_*ij*_ is the distance between beads *i* and *j*, the cutoff distance *r*_cut_ = 2^1/6^σ corresponds to the minimum of the 12–6 Lennard-Jones potential, and the interaction amplitude is set equal to the thermal energy of the system, ϵ = *K*b*T*.

Bonds between consecutive monomers in a ring were modeled via a finitely extensible nonlinear elastic (FENE) potential^50^
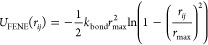
2where *r*_*ij*_ is the distance between bonded monomers *i* and *j*, *r*_max_ = 3σ is the maximum allowed bond elongation, and *k*_bond_ = 30 *K*b*T*/σ^2^. This parametrization, which differs from the Kremer-Grest one^50^ for the larger value of *r*_max_, results in a probability distribution of intraring bond lengths that is largely independent of whether the consecutive monomers are neutral or charged, (i.e., whether they additionally interact via the Coulomb term described below), see Figure S1.

Electrostatic interactions between two charged particles *i* and *j* were accounted for with a Coulomb potential,
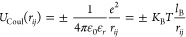
3and were computed using the P^3^M method, a hybrid method combining direct short-range and mesh-based long-range force calculations,^51^ with an accuracy of 10^–3^.^52^ In eq 3, *e* is the elementary charge, *l*b is the Bjerrum length, *ε*_0_ is the vacuum dielectric constant, *ε*_*r*_ is the relative permittivity of the medium, and the ± prefactor reflects whether the particles carry charges with the same or opposite sign. The solvent is implicitly represented by a uniform dielectric continuum, as is common in primitive models of electrolytes. Setting σ = 3.55 Å and *L*_box_ = 121.23σ results in a monomer concentration of 10^–2^ mol/L and a Bjerrum length *l*_B_ = 2σ = 7.10 Å, the latter a value typical for diluted aqueous solutions of polyelectrolyte at room temperature.

The system was evolved via Langevin dynamics simulations in the canonical ensemble at *T* = 298 K with default values^50^ for the friction coefficient, γ, and particles’ mass, *M*. The dynamics was integrated with a velocity Verlet algorithm using a time step δ*t* = 0.01τlj, where  is the characteristic Lennard-Jones time. Simulations were performed using the Molecular Dynamics software package ESPResSo v4.1.^53^ To avoid system instabilities and the rupture of the mechanical links due to Coulomb interactions, the system has been equilibrated as follows: after 100τ_LJ_ with electrostatics switched off, Coulomb interactions were gradually introduced by increasing the nominal ionization degree α of the charged monomers from 0 to 100% by means of a constant pH approach,^54,55^ in which the nominal pH was increased from −10 (α = 0) to 10 (α = 1) by ΔpH = 2 every 100τlj; then, the fully charged system was further equilibrated for 2 × 10^4^τlj.

The neutral block length was varied from *m*_neu_ = 0 (i.e., fully charged rings) to *m*_neu_ = *m* = 20 (fully neutral rings). For each value of *m*_neu_, we equilibrated the system and ran 5 independent simulations of duration 10^5^τlj, sampling configurations at 10τlj intervals.

### Observables

To characterize the catenane’s properties across different scales, we computed canonical expectation values of various observables by taking averages over the sampled conformations.

Going from local to global metric observables, we considered: (i) the root mean squared gyration radius of individual rings, ; (ii) the average distance of the centers of mass of neighboring rings, i.e., the mechanical bond length, *b*; (iii) the distance of minimum approach of concatenated rings *A* and *B*, defined as the minimum distance between any monomer in ring *A* and any monomer in ring *B*, i.e., *d*_min_ = min_*i*∈*A*,*j*∈*B*_*r*_*ij*_, and (iv) the charged/neutral character of the contacting monomers; the correlation of (v) mechanical bond vectors and (vi) the chemical orientation vectors *v̂*^cn^, defined as the (normalized) distance vectors between the midpoints of the charged and neutral blocks; (vii) the gyration radius and (viii) and end-to-end distance of the entire catenane’s backbone.

For the characteristic times of the catenane’s internal dynamics, we considered the reorientation time, that is, the decay time of the orientational correlation function of the normalized end-to-end vector, *R̂*_ee_,
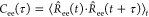
4where τ is the time lag and ⟨⟩_*t*_ denotes the time average. The characteristic reorientation time of the catenane, , was obtained by integrating *C*_ee_(τ) from τ = 0 up to the smallest value of τ for which the correlation falls below 0.01.

For the relaxation dynamics at the local scale, we considered the characteristic reorientational time^56^ of the central ring, . As customary,^57^ this was computed by averaging the orientational correlation functions of all its normalized diameter vectors.

## Results

We first examined how the composition of the co-PE rings and their mechanical bonding influence the catenane metric properties across multiple scales.

### Overall Catenane Size

**Figure 2:**
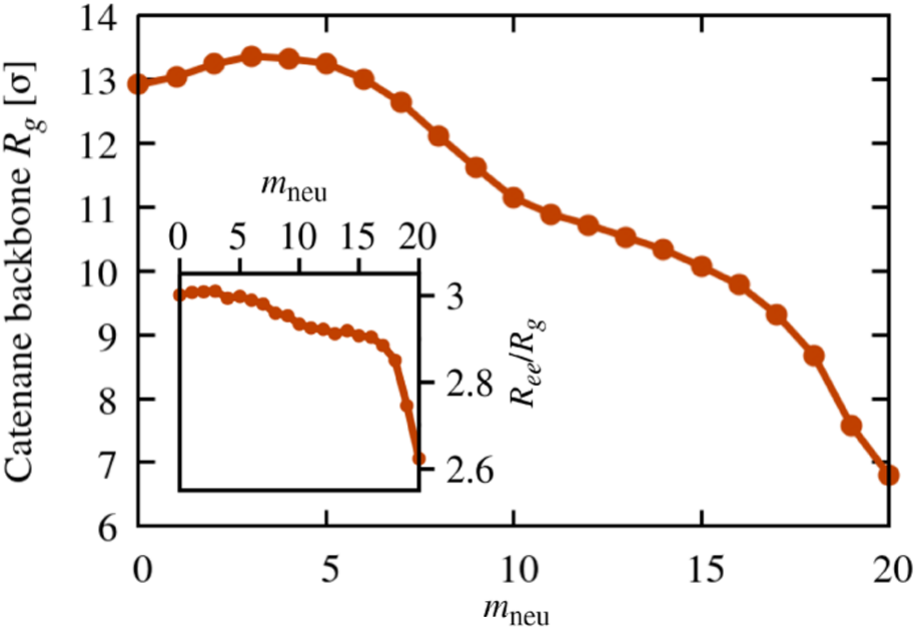
Gyration radius of the catenane backbone, *R*_g_, as a function of ring composition, *m*_neu_. The inset shows the dependence of the catenane’s gyration radius to end-to-end distance ratio, *R*_ee_/*R*_g_, on the ring composition.

We first analyzed the dependence on ring composition of the overall size of the catenane, which we measured via the root mean square values of the gyration radius, *R*_g_, and end to end distance, *R*_ee_, of the catenanes’ backbone, as sketched in Figure 1. The data for *R*_g_, are shown in Figure 2 and highlight two notable properties. First, *R*_g_, has a nonmonotonic dependence on *m*_neu_, with a maximum at *m*_neu_ = 3. The *R*_g_ fluctuations are similarly nonmonotonic (Figure S2). Second, the *R*_g_ curvature changes sign twice, with two concave regions flanking an intermediate convex one for 7 ≲ *m*_neu_ ≲ 15. Analogous properties hold for the root mean squared end-to-end distance, *R*_ee_, which behaves similarly to *R*_g_ except for the milder decrease for *m*_neu_ > 15, see Figure S3 and the inset of Figure 2. It is interesting to compare the dependence of the overall catenane size on the co-PE ring composition with that observed in mechanically bonded block copolymer rings with rigid and flexible segments. These systems have recently been studied in the context of two-dimensional chainmails, where the average size varied monotonically with ring composition.^19^ Although the systems’ dimensionalities differ, this result and others presented later suggest that the nonmonotonic behavior of *R*_g_ and *R*_ee_ is specific to charged/neutral copolymers.

### Rings’ Size and Compenetration

**Figure 3:**
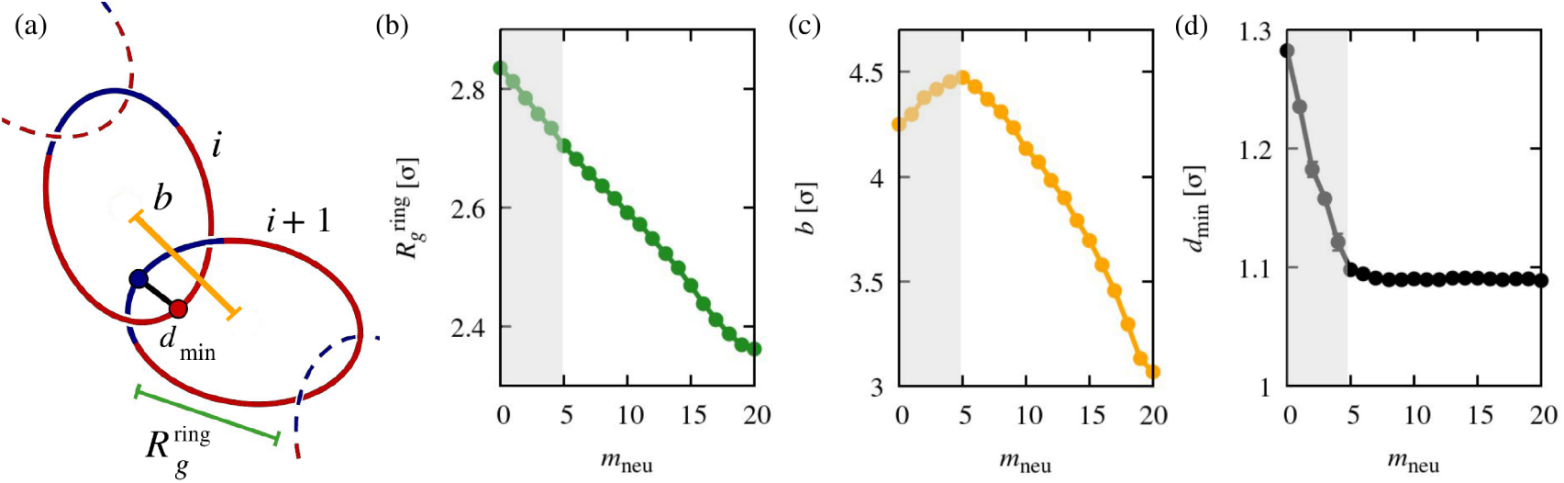
Local metric properties of catenanes as a function of ring composition. The profiled observables are the average ring’s gyration radius, ; the average mechanical bond length, *b* (defined as the distance between the centers of mass of concatenated rings); and the average distance of minimum approach between monomers of two concatenated rings, *d*_min_, see sketch in panel (a). The shaded band marks the interval 0 ≤ *m*_neu_ ≤ 5 where the *b* and *d*_min_ curves exhibit qualitatively distinct behavior compared to longer neutral blocks.

Turning to the local metric properties, we then considered the size and degree of compenetration of concatenated rings. To this end, we analyzed the root mean squared gyration radius of individual rings, , the average distance of minimum approach of linked rings, *d*_min_, and the average distance of their centers of mass, also termed mechanical bond length, *b*, see sketch in Figure 3a. The dependence of these observables on *m*_neu_ is illustrated in panels b–d of Figure 3.

Compared to the entire catenane, the gyration radius of individual concatenated rings has a different *m*_neu_ dependence. In fact, it decreases monotonically and without significant changes in curvature. The decrease of  with *m*_neu_ reflects the diminishing intraring electrostatic repulsion, which maintains rings in an approximate planar circular state (Figure S7). Notice that  decreases by only about 15% going from fully charged to fully neutral rings, whereas the catenane’s *R*_g_ decreases by nearly a factor of 2.

Interestingly, Figure 3c reveals a nonmonotonicity of the mechanical bond length, *b*, which has a maximum at *m*_neu_ = 5. Although the peak location is similar to that of the catenane’s *R*_g_, the *b* profile maintains the same curvature, unlike in the *R*_g_ case.

Finally, Figure 3d shows the profile of the average distance of minimum approach of monomers in mechanically linked rings, *d*_min_, which we use as a measure of mechanical bond tightness. For *m*_neu_ = 0, *d*_min_ is equal to 1.3σ, indicating that the inter-ring electrostatic repulsion, though lessened by the present counterions, prevents fully charged concatenated rings from being in tight contact. As the length of the neutral block increases, *d*_min_ decreases and eventually plateaus for *m*_neu_ > 5 at 1.09σ. This value is approximately equal to the distance of bonded monomers in a ring; see Figure S1. This indicates that tight concatenation can already occur at *m*_neu_ = 5, even though the rings are still mostly charged at this composition.

Overall, the results of Figure 3 point at two distinct regimes for the local metric properties. For *m*_neu_ > 5, consecutive rings are in tight contact and their size and center of mass distance decrease significantly as the fully neutral case is approached. Instead, even though counterions are present for *m*_neu_ < 5, the stronger electrostatic repulsion makes rings larger and keeps them more separated along the catenane’s backbone.

The implications of the two effects for the nonmonotonicity of *b* is aptly conveyed by the following heuristic argument. Consider an idealized catenane composed of elliptic rings, with their longest axes aligned to the catenane backbone and lying in alternating orthogonal planes. Decreasing the rings’ size,  via an affine transformation of the elliptic rings, clearly shortens the mechanical bond length *b*. Conversely, decreasing *d*_min_ while keeping  constant causes *b* to increase. Thus, reducing  and *d*_min_ has opposite effects on *b*. The nonmonotonicity of *b*(*m*_neu_) is precisely caused by such competition, since both  and *d*_min_ decrease with *m*_neu_ (Figure 3b,d). This point is quantitatively illustrated in Figure S4, which shows the *m*_neu_ dependence of the approximate mechanical bond length, *b*_approx_= 2*l*_1_ – *d*_min_, where *l*_1_ is the square root of the largest eigenvalue of the average gyration tensor of individual rings.

The discussed interplay of *b* and  provides an apt illustration of how global properties of the co-PE polycatenane are tied to local features underpinned by mechanical bonding.

### Relative Orientation of Neighboring Rings

**Figure 4:**
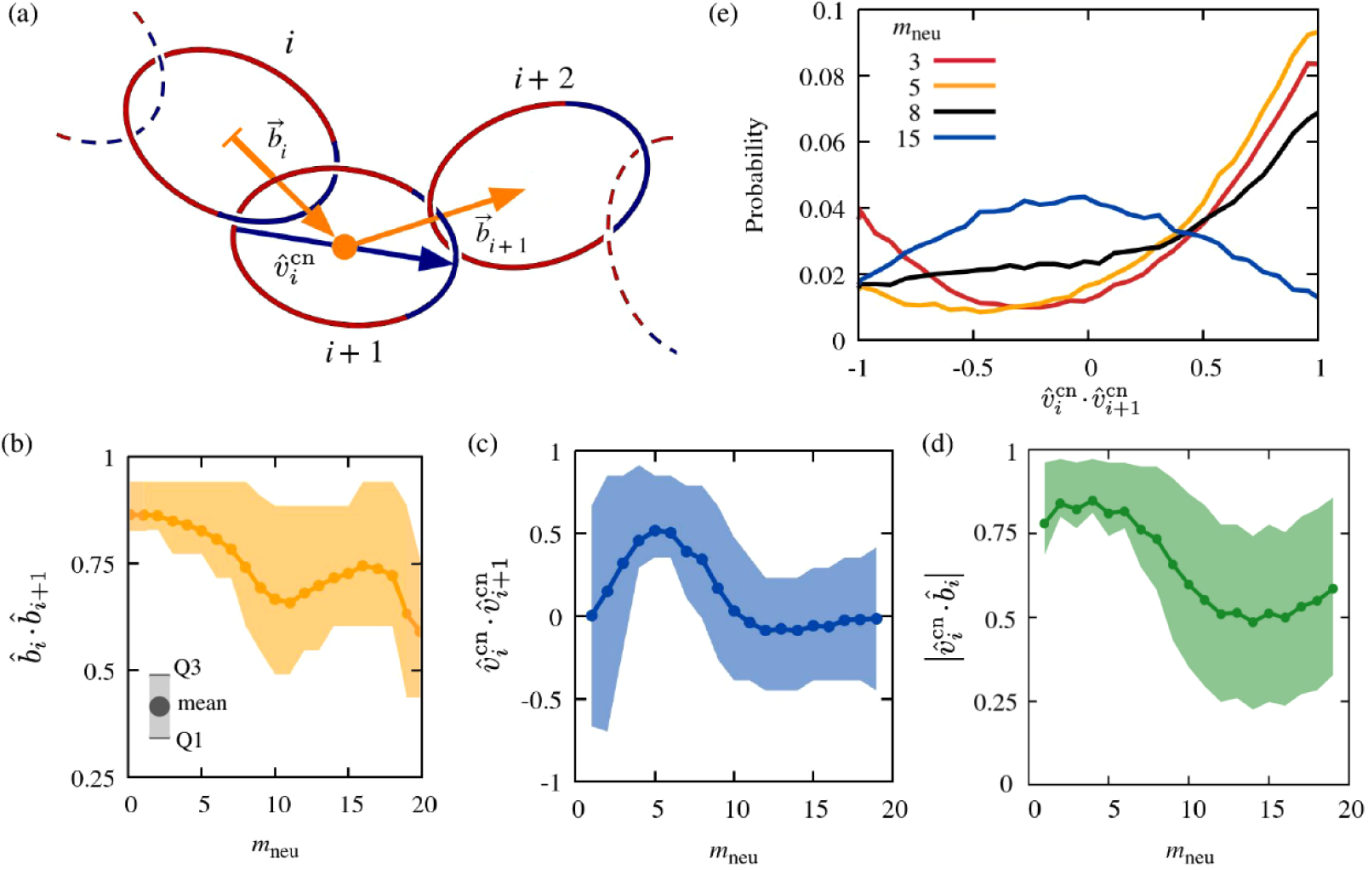
Internal orientational correlation of catenanes as a function of ring composition. The profiled observables involve scalar products of mechanical bond vectors, *b̂*, and charged-to-neutral vectors, *v̂*^cn^; see sketch in panel (a). Panels (b–d) show the *m*_neu_ dependence of the orientational correlation of mechanical bonds and chemical orientation vectors for the same and consecutive rings. Data points represent the average scalar product value, while the shaded band indicates the Q1–Q3 interquartile range, spanning from the 25th (Q1) to the 75th (Q3) percentiles. Panel (e) shows the probability distributions for consecutive charged-to-neutral vectors at different *m*_neu_ values.

Next, we studied the relative orientation of concatenated rings and their neutral and charged blocks. Specifically, we analyzed the orientational correlations of mechanical bonds and the vectors connecting the midpoint monomers of the charged and neutral blocks on the same ring. For brevity, we shall refer to the latter as the chemical orientation vector, or charged-to-neutral vector, and indicate it as *v⃗*^cn^; see the sketch in Figure 4a.

As a measure of the effective rigidity of the catenane, we used the average scalar product of consecutive mechanical bonds, *b̂*_*i*_ · *b̂*_*i*+1_, where the ^^^ symbol denotes that the vectors are normalized to unit length. To cover the possible combinations of mechanical bonds and charged-to-neutral vectors, we additionally analyzed the average scalar products  · *b̂*_*i*_, and .

The dependence of these various measures of orientational order on ring composition is illustrated in Figure 4b–d.

The data in panel (b) show that *b̂*_i_ · *b̂*_*i*+1_ remains above 0.5 for all values of *m*_neu_. This indicates that consecutive mechanical bonds have a strong alignment correlation across all ring compositions; see also Figure S8. In spite of this, it also emerges that *b̂*_i_ · *b̂*_*i*+1_ has a minimum for intermediate values of *m*_neu_. Specifically, the alignment correlation decreases from a maximum of 0.86 for *m*_neu_ = 0 (fully charged rings) to 0.66 for *m*_neu_ = 10 (50–50 ring composition). Beyond this local minimum, *b̂*_i_ · *b̂*_*i*+1_ rises to 0.75 for *m*_neu_ = 16, before dropping to the global minimum of 0.6 for fully neutral rings, *m*_neu_ = 20. This establishes the unexpected result that the effective bending rigidity of the catenane has a nonmonotonic dependence on the length of the neutral/charged segments. The corresponding *m*_neu_ dependence of the effective persistence length of the catenane backbone is presented in Figure S5. The same figure shows that the nearly 2-fold variation in catenane size with *m*_neu_ (Figure 2) is approximately captured by a Kratky–Porod model informed by the catenane’s persistence and mechanical bond lengths.

Panel (c) of Figure 4 shows that an opposite nonmonotonicity is present for . Note that the average scalar product of consecutive chemical orientation vectors is approximately zero for *m*_neu_ equal to 1 and 19, which is close to the cases of uniformly charged/neutral rings, where *v̂*^cn^ cannot be defined. The nonmonotonic curve bridging these limiting cases is strongly asymmetric with respect to the 50–50 composition. For mostly charged rings, *m*_neu_ < 10, the average scalar product is unimodal, peaking at 0.5 for *m*_neu_ = 5, while for mostly neutral rings it remains close to zero. The data thus indicate that the ″chemical orientation″ correlation of neighboring rings is strongest at *m*_neu_ = 5 and negligible for *m*_neu_ > 10.

This result is best understood by considering the probability distributions for the scalar products  at different ring compositions, which are shown in panel (e). The distribution is bimodal for *m*_neu_ = 3, with maxima corresponding to parallel and antiparallel chemical alignments. The parallel alignment is the dominant one and becomes even more so for *m*_neu_ = 5, yielding the highest average scalar product. As *m*_neu_ increases to 8, the statistical weight of the parallel order diminishes as intermediate negative values of the scalar product become populated, too. Finally, for *m*_neu_ = 15, the curvature of the probability distribution of the scalar products changes sign. The parallel and antiparallel states become the least populated ones, while the distribution becomes broad and approximately symmetric with respect to zero. The same holds throughout the *m*_neu_ > 10 interval, thus accounting for the observation in panel (c) of the flattening to zero of the  curve for 12 ≤ *m*_neu_ ≤ 19.

Finally, we discuss the relative orientation of the normalized chemical vector of a ring and one of its two mechanical bonds. We note that while the chemical orientation of individual rings is uniquely defined, the mechanical backbone is not oriented. Thus, inverting the orientation of all mechanical bonds *b̂*_*i*_ → −*b̂*_*i*_ provides a legitimate and entirely equivalent description of the catenane backbone, see also Figure S6. To discount such inversion symmetry, we thus considered the modulus of the scalar product, . The data, which are plotted in panel (d), indicate that  and *b̂*_*i*_ are approximately parallel for *m*_neu_ ≤ 5. As the neutral segment length increases, the good alignment of the chemical orientation vectors and the local backbone is progressively lost. In fact, throughout *m*_neu_ ≥ 10 the average value and spread of  are close to those expected for two randomly oriented vectors, a condition practically realized as the fully neutral case is approached.

All considered, the results of Figure 4b–d establish that (i) even small charged segments of as few as four monomers (*m*_neu_ = 16) endow the catenane backbone with an effective bending rigidity that is significantly larger than the fully neutral case (*m*_neu_ = 20), (ii) chemical orientation vectors are well aligned to the backbone for *m*_neu_ ≤ 5, and (iii) the chemical orientation of neighboring rings is most correlated at *m*_neu_ = 5, i.e., at the crossover between the two local metric regimes discussed in connection with Figure 3.

### Chemical Orientation of Distant Rings, and Emerging Defects

To complete the analysis, we considered the correlation of chemical orientation vectors of ring pairs, *i* and *j*, at increasing backbone (sequence) distance, |*i* – *j*|. To avoid end effects, the first and last rings in the catenanes were not considered, so that the analysis is performed for |*i* – *j*| ranging from 1 to *n* – 3 = 9.

**Figure 5:**
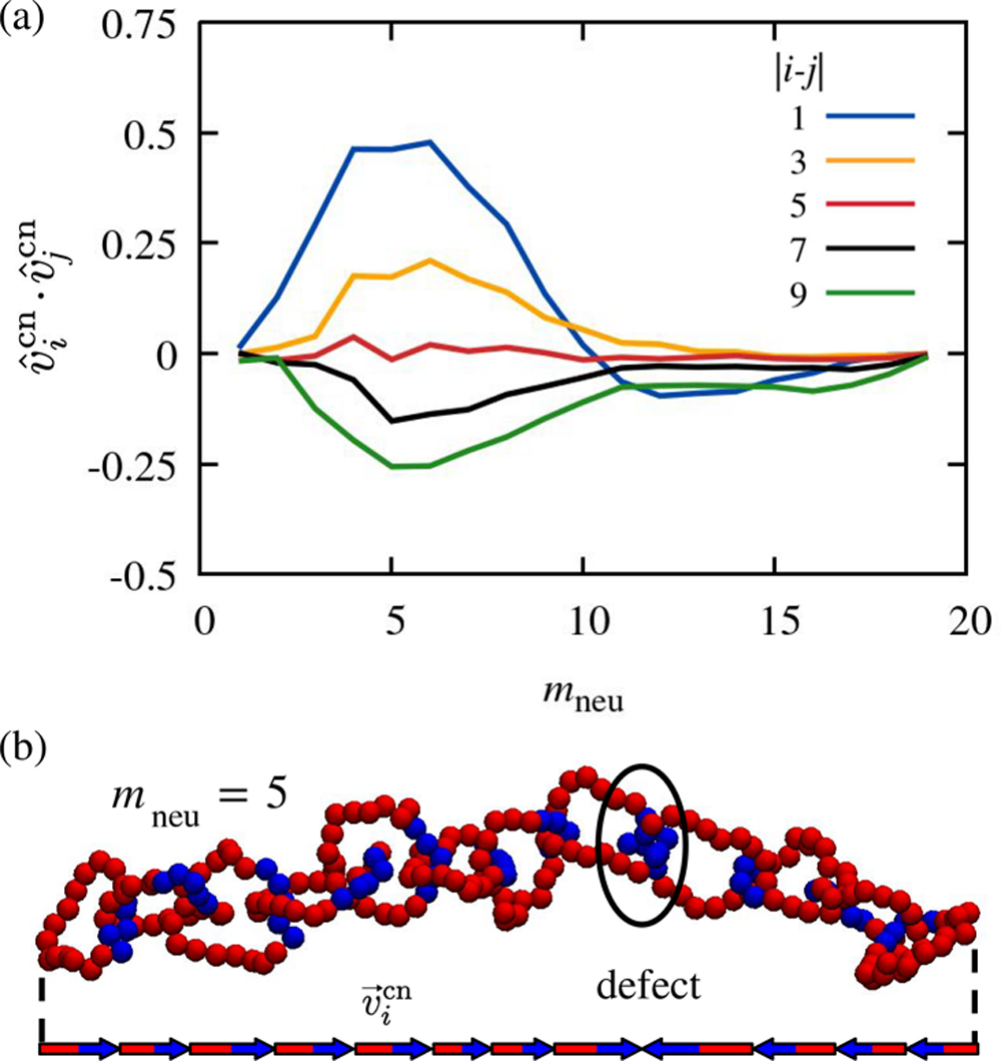
Orientational correlation defects. Panel (a) shows the *m*_neu_ dependence of the orientational correlation (average scalar product) of pairs of charged-to-neutral vectors, *v⃗*^cn^, at various distances along the catenane backbone. Negative values of the average scalar products are due to chemical orientation defects such as the one illustrated in panel (b) for a typical configuration at *m*_neu_ = 5. The arrows sketched at the bottom represent the projections of the charged-to-neutral vectors along the end-to-end distance vector of the catenane. The defect separates oppositely oriented runs of the *v⃗*^cn^ projections.

The results are shown in Figure 5. The average scalar products  remain close to zero for *m*_neu_ > 10 at all backbone distances. This indicates that no significant chemical orientational correlation exists at any sequence separation between rings that are mostly neutral. We recall that in this same *m*_neu_ range, the *v⃗*^cn^ vectors are approximately randomly oriented with respect to the local backbone, too.

However, when the rings are mostly charged, a qualitative change occurs for increasing sequence separations. The data for the smallest possible distance, |*i* – *j*|=1, correspond to the same neighboring rings already discussed in Figure 4c, where the largest orientational correlation (+0.5) occurs at *m*_neu_ = 5. As |*i* – *j*| increases, the correlation peak gradually diminishes, flattens to zero, and eventually turns into a negative peak, indicative of anticorrelation. Interestingly, the positive and negative peaks all occur near *m*_neu_ = 5. The most negative correlation is about −0.25, i.e., half the magnitude of the largest positive one.

The results establish that, for the 3 ≲ *m*_neu_ ≲ 8 compositions, the chemical orientation of distant rings is typically antiparallel, whereas that of neighboring rings is mostly parallel. This indicates that at these ring compositions catenanes are likely to contain defects, i.e., antiparallel pairs of consecutive charged-to-neutral vectors within a sequence of parallel-oriented pairs.

We recall that in this *m*_neu_ range, the chemical orientation vectors are mostly aligned along the mechanical backbone that, in turn, has a high effective rigidity (Figure 4). These considerations suggest that a convenient representation of the defects can be achieved by considering the projections of the chemical orientation vectors along the catenane’s end-to-end vector. The illustration of one such defect is provided in Figure 5b, where it is noticed that the projected  vectors change directionality at the interface of rings *i* = 8 and *i* = 9. The resulting orientation pattern is reminiscent of the energetically costly domain walls in one-dimensional Ising chains. However, the analogy is only qualitative because, differently from Ising chains, domain walls in ordered PE-catenanes (featuring N–C interfaces only) can be introduced in different ways: either via a C–C interface, which is typically energetically costly, or via an N–N interface, as shown in Figure 5b.

The systematic presence of isolated defects for *m*_neu_ ∼ 5 is underscored by the fact that the most probable orientations of consecutive chemical vectors are the parallel and antiparallel ones (Figure 4e). Detailed analysis, presented in the next subsection and in Figure S13, reveals that at the considered catenane’s length (*n* = 12), the most probable number of defects for *m*_neu_ ∼ 3–5 is 1, although 0 and 2 defects have a sizable probability, too.

As evident from Figure 5b, the emergence of defects is associated with two neutral blocks interfacing at the mechanically bonded region.

### Patterns of Contacting Monomers

To investigate the microscopic basis of the chemical orientational correlation, we analyzed the contact probability between charged and neutral monomers of neighboring rings as a function of ring composition. Our investigation involved two complementary approaches: first, we determined which contour regions of a ring are closest to the concatenated neighbors; next, we examined the contact probability between charged and neutral blocks of concatenated rings.

**Figure 6:**
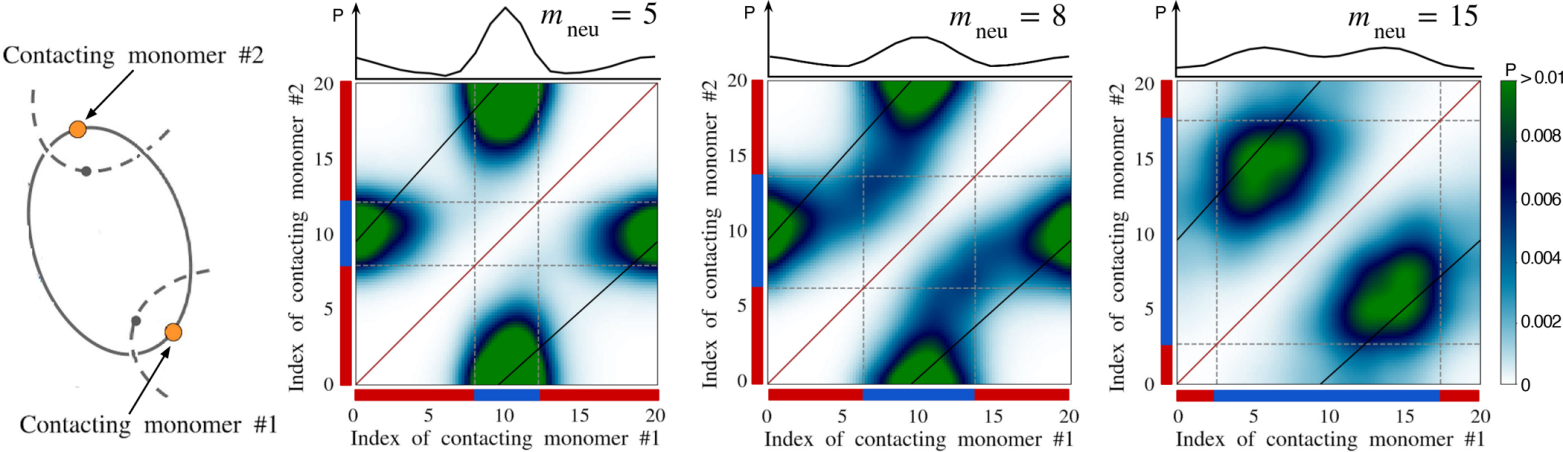
Probability distribution of mechanically bonded monomers in a given ring. The heatmaps represent the joint probability distribution of the indices of the two monomers of the central ring in the catenane closest to each neighboring ring, as sketched on the left. The heatmaps are shown for three different values of *m*_neu_, with the neutral (blue) and charged (red) character of the monomers indicated by the colored sidebars. The profile at the top is the marginalized (one-dimensional) probability distribution. The diagonal symmetry of the heatmaps, reflecting the equivalence of the two monomers, was not imposed on the data.

For the first analysis, we focused on the central ring in the catenane and identified the two monomers closest to the neighboring rings, as illustrated in the sketch in Figure 6. By repeating this procedure across the sampled conformations, we computed the joint probability distribution of the indices of the two mechanically bonded monomers. The results for various ring compositions are shown in Figure 6 as two-dimensional heatmaps with periodic boundary conditions; analogous plots for additional values of *m*_neu_ are shown in Figure S11. The diagonal symmetry was not imposed *a priori* and reflects the statistical equivalence of the two neighboring rings. The color-coded sidebars denote the monomer types (charged or neutral), whereas dashed lines indicate the block boundaries. The top plots provide one-dimensional projections of the two-dimensional probability distributions and capture the probability that specific monomers within the neutral or charged blocks are in contact with one of the neighboring rings.

The density plot for *m*_neu_ = 5 reveals that mechanical bonding predominantly involves monomers located at the center of the neutral and charged blocks, thus corresponding to diametrically opposite positions on the ring contour. Interestingly, monomers close to the block boundaries are seldom in contact with the neighboring rings.

Two qualitative changes emerge with slightly longer neutral segments, *m*_neu_ = 8. On the one hand, the boundary regions of the charged blocks become increasingly likely to participate in mechanical bonding. On the other hand, a significant probability density appears in the heatmap region where both contact points reside on the neutral segment, thus evidencing that the *m*_neu_ = 8 segment is sufficiently long to occasionally accommodate mechanical bonds with both neighboring rings simultaneously, despite all rings being predominantly charged and therefore repelling one another. Due to this repulsion, the two contact points are at opposite edges of the neutral segment. This property makes the *m*_neu_ = 10 composition consequential for various metric observables, as well as dynamical ones described later, that attain their local/global extremal values at the balanced composition. The significance of *m*_neu_ = 10 is underscored by the analysis of the fraction of condensed counterions and the radial distribution functions of the system (see Figures S9 and S10). While the latter do not deviate from what commonly expected, the former exhibits a distinct regime shift at this nearby compositions.

These findings clarify key aspects of the considered system; in fact, the change in trend for the number of condensed counterions in Figure S10 could, in principle, be attributed to the decrease in probability that a charged segment on a ring sits relatively close to, or directly facing, a similar feature on another, mechanically linked, moiety. If so, the probability of counterions condensing “between two rings” (i.e., forming electrostatic bridges) due to the locally high electrostatic potential should also decrease, as it happens in knotted copolyelectrolytes.^58^ For the latter, we have already highlighted that essential crossings tend to foster a higher counterion condensation than the average,^35^ especially when in the presence of divalent ions. Importantly, the origin of the divalent species (i.e., whether coming as chain’s counterions or due to added salts) appears irrelevant.

This contact mode becomes dominant and typical as the neutral segment length increases, as illustrated by the *m*_neu_ = 15 heatmap. In this case, the one-dimensional profile at the top reveals that contacts involving the central monomers of the blocks are suppressed, particularly in the charged region. Instead, the most probable contacting monomers are situated near the edges of the neutral block.

In the second type of analysis, for each pair of interlocked rings, we identified the type – neutral (N) or charged (C) – of the two closest monomers, one from each ring. We recall that these pairs correspond to those defining *d*_min_ in Figure 3d. We then calculated the total numbers of N–N, N–C, and C–C contacting pairs in the catenane and averaged them over the sampled conformations.

**Figure 7:**
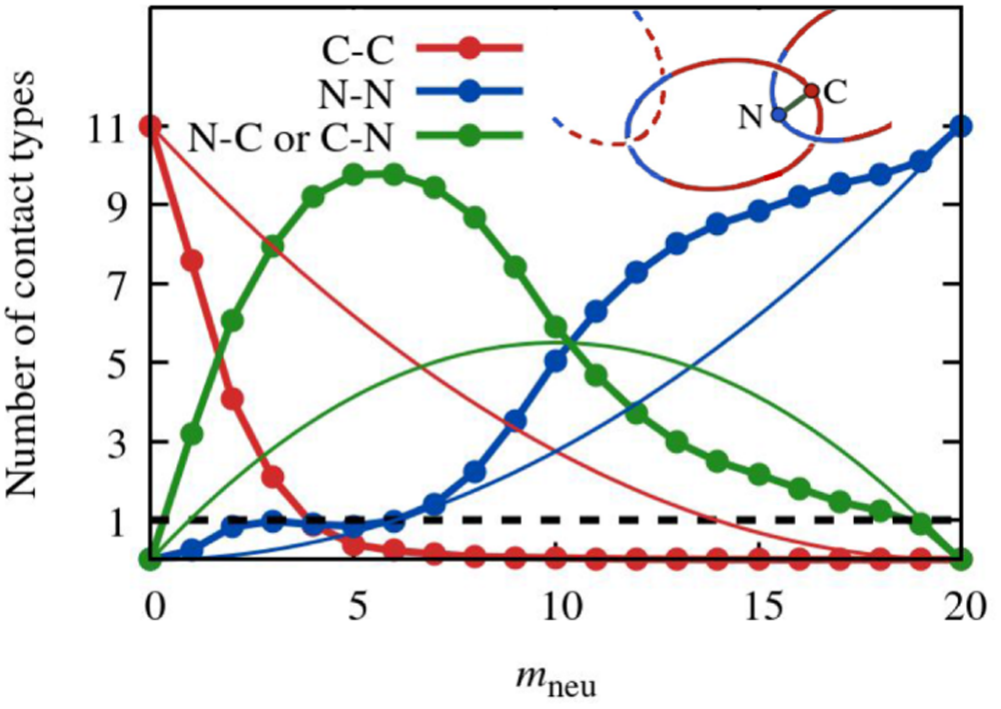
Average number of contacting pair types in the catenane as a function of ring composition. The curves show the *m*_neu_ dependence of the average number of neutral–neutral (N–N), charged–charged (C–C), and mixed (C–N or N–C) pairs of contacting monomers in the catenane. The contacts refer to the two closest monomers of concatenated rings. The average numbers of contact types sum to *n* – 1 = 11 at each *m*_neu_ value. The three colored thin curves represent mean-field-like approximations, see main text. For 2 ≤ *m*_neu_ ≤ 7, the N–N curves plateau at about the value of 1, marked by the dashed horizontal line.

The resulting curves are shown in Figure 7, plotted as a function of *m*_neu_. Analogous data, but referred to the contacts types probabilities of individual mechanical bonds are shown in Figure S12. The data in Figure 7 reveal that C–C contacts decrease rapidly with the introduction of even a small number of neutral monomers and become negligible already at *m*_neu_ = 5, despite the charged block being three times longer than the neutral one. The fact that the trend of the C–C curve parallels that of the minimum approach distance of concatenated rings (*d*_min_ curve in Figure 3d) suggests that mechanical links become taut for *m*_neu_ > 5.

For 2 ≤ *m*_neu_ ≤ 10, C–N contacts are prevalent. Note that the peak in C–N contact number does not occur for the half-charged/half-neutral composition, but rather at *m*_neu_ = 5, and N–N contacts become dominant for *m*_neu_ > 10. Interestingly, for 2 ≤ *m*_neu_ ≤ 7, the N–N curve plateaus near the value of 1. Consistently with the data of Figure 5, this indicates that, on average, there is one chemical orientation defect in the catenane. Indeed, detailed analysis of the distributions of contacting pairs type shows that, for *m*_neu_ = 3–5, *m* = 12 catenanes are likely to feature from 0 to 2 defects, the single defect case being the most probably by far; see Figure S13.

For reference, in Figure 7 we also included baseline contact numbers (thin lines) computed from mean-field–like (MF) pairing combinatorics. The expressions, based on the fraction of neutral and charged monomers in a ring, are
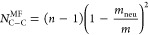
5
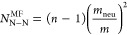
6
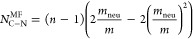
7

All observed contact numbers deviate significantly from the MF estimates, with the largest discrepancy observed for the C–N case. The actual C–N peak at *m*_neu_ = 5 is substantially shifted from the MF one (peaking at *m*_neu_ = 10), and its value is nearly twice the MF one. As the neutral segment length increases, C–C contacts decay faster than predicted by the MF model. Finally, throughout the *m*_neu_ > 10 interval, N–N contacts are over-represented compared to the MF curve, which also lacks the plateau associated with the chemical orientation defect.

Finally, we note that the curves for all three contact types in Figure 7 differ markedly from those observed in the aforementioned chainmails of copolymer rings with rigid and flexible segments.^19^ The latter are satisfactorily approximated by the MF curves, which exhibit mirror symmetry about the 50–50 composition, corresponding to an interchange of the two segment types. Conversely, the curves in Figure 7 are strongly asymmetric because electrostatic interactions introduce fundamental differences when neutral and charged monomers are interchanged. As a result, mechanically bonded systems made of co-PEs and neutral diblock copolymers have starkly different properties, reinforcing and generalizing the inequivalence previously established for conventionally bonded co-PEs and uncharged block copolymers.^44,59^

### Dynamics

We now turn to the effects of the ring composition on the catenanes’ dynamics across different scales.

For the global dynamics, we analyzed the characteristic rotational time of the catenane, , which is defined as the correlation time of the end-to-end vector orientation; see Methods. This global relaxation mode was chosen for analysis because it decays much more slowly than the autocorrelation of other standard metric observables, such as the radius of gyration; see Figure S14.

**Figure 8:**
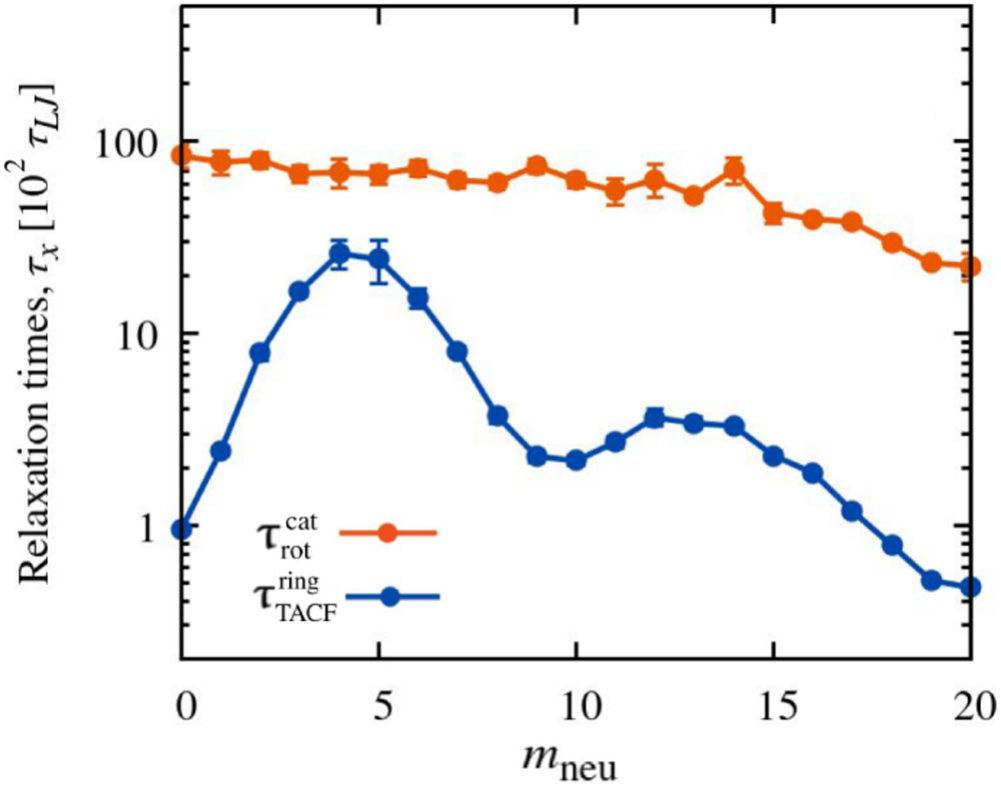
Characteristic times of global and local relaxation modes as a function of ring composition. The curves show the *m*_neu_ dependence of the characteristic rotational times of the entire catenane and one of its rings. The former, , is the characteristic decay time of the orientational correlation function of the catenane end-to-end vector. The latter,  is the characteristic decay time of the orientational correlation function of the ring’s diameter vectors, averaged over all diameters.

Figure 8 shows that  decreases steadily with *m*_neu_, dropping by approximately a factor of 3 from fully charged to fully neutral rings. The reduction is consistent with the relaxation becoming more rapid as the catenane shrinks with increasing *m*_neu_ (Figure 2) to the reduced electrostatic repulsion. Indeed,  and  are approximately proportional, analogous to the behavior of Rouse-like models in the absence of hydrodynamic effects, see Figure S16. Interestingly, the rotational relaxation time of mechanical bond vectors is smaller but still comparable to  throughout the entire *m*_neu_, see Figure S14. This is consistent with the observed overall stiffness of the catenane’s backbone at the considered length, *n* = 12.

Differently from the global relaxation dynamics, the internal dynamics of individual rings exhibit a more varied and complex dependence on *m*_neu_. To characterize it, we analyzed the characteristic reorientational time^56^ of one of the two central rings, , which we computed by averaging the rotational autocorrelation functions of all its diameter vectors;^57^ see Methods.

Notably, Figure 8 shows that the  (*m*_neu_) curve is bimodal, peaking at *m*_neu_ ≈ 5 and *m*_neu_ ≈ 15 and with an intervening local minimum at *m*_neu_ = 10. Across most of the *m*_neu_ range,  is one to 2 orders of magnitude smaller than . An exception is *m*_neu_ ≈ 5, where  is of the same order as .

The  profile is best discussed starting from the limiting cases of fully charged and fully neutral rings.  decreases by about a factor of 2 going from the former to the latter case. In relative terms, this slowdown is similar to that of the global rotational time, . However, due to the differing length scales of these two modes,  is approximately 2 orders of magnitude shorter than .

The fact that local relaxation is significantly slower at *m*_neu_ = 5 compared to fully charged rings reflects the particular structural organization of the catenane at this composition. As noted in connection with Figure 3c,d, the *m*_neu_ = 5 neutral blocks are already sufficiently long to allow concatenated rings to form tight mechanical bonds at the charged-neutral clasped interfaces Figure 3c,d. At the same time, the electrostatic repulsion at *m*_neu_ = 5 maintains a high rigidity of the catenane backbone while also locking their chemical alignment of neighboring rings (4b,c). These local geometrical constraints hinder the reorientational motion of the rings and reflect in their noticeably slow relaxation at *m*_neu_ = 5.

As *m*_neu_ is increased from 5 to 10, both the backbone rigidity and chemical alignment of neighboring rings decrease. As a result, the rings gain rotational freedom relative to each other without compromising the favorable charged-neutral interlockings. This reflects in the decrease of  in the same *m*_neu_ range. At *m*_neu_ = 10, the neutral segments become large enough to be co-opted in the mechanical bonding with both neighboring rings. At *m*_neu_ ≈ 10, where N–N contacts dominate and the alignment between *v*^cn^ and the catenane backbone is lost, a distinct  regime emerges. Moving toward the fully neutral case, the widening gap between  and  highlights the progressive decoupling of the ring’s internal dynamics and the global dynamics of the catenane.

We conclude the analysis by discussing the dynamics of the chemical orientation defects. To this end, we examined the time evolution of the orientation of consecutive charged-to-neutral vectors relative to their local backbone direction, quantified by the scalar products .

**Figure 9:**
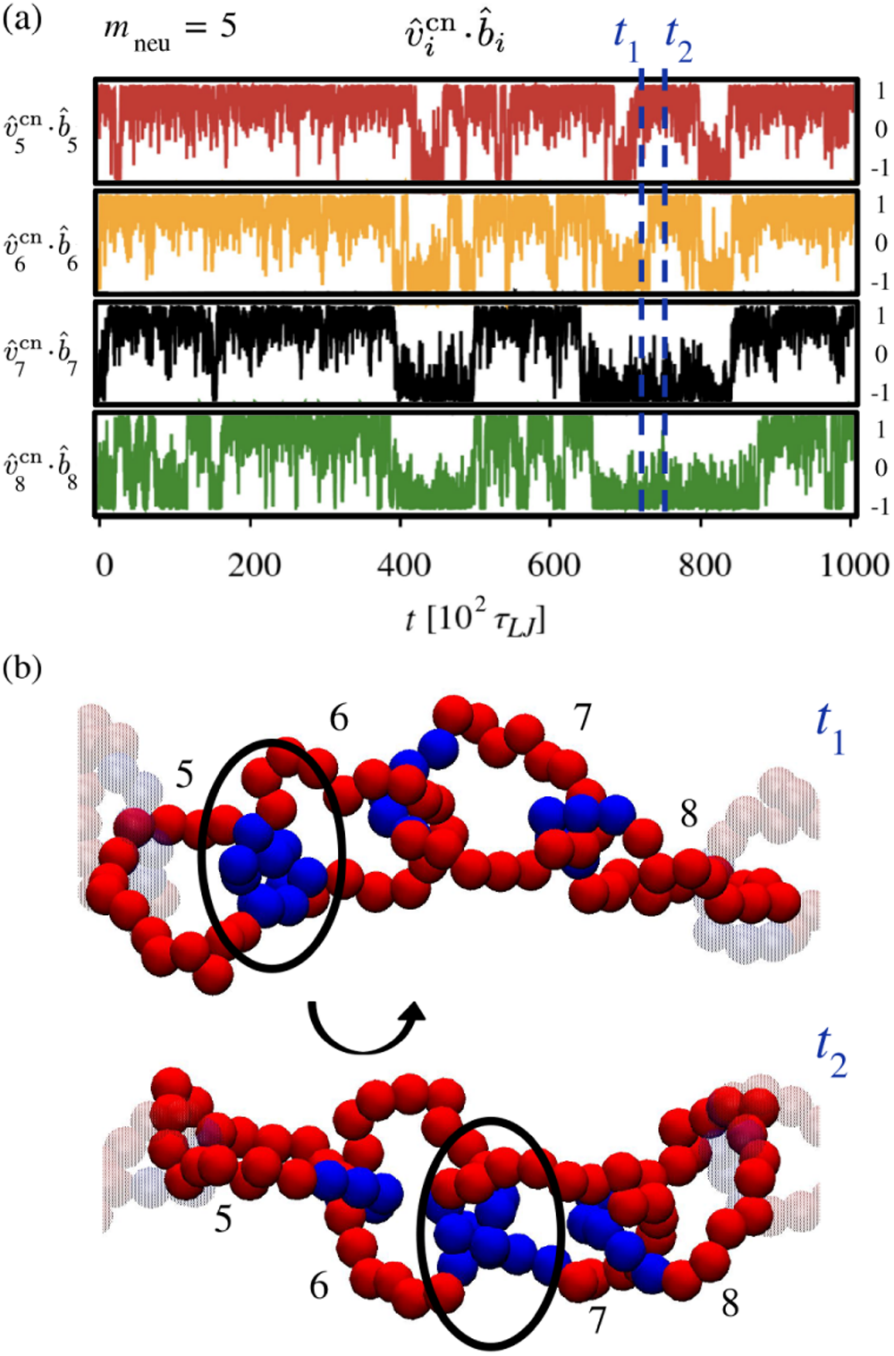
Dynamics of chemical orientation defects. The traces in panel (a) represent the time evolution of the orientational correlation (scalar product) of consecutive charged-to-neutral vectors,  and one of the two corresponding mechanical bonds *b̂*_*i*_. The data are for a stretch of a few consecutive rings at *m*_neu_ = 5. The configurations corresponding to the two selected times (dashed lines) are shown in panel (b) and illustrate the hopping of the defect across neighboring rings.

Figure 9a shows typical traces of the scalar products over a timespan of 10^5^τlj, several times longer than the global relaxation time . The data refer to four consecutive rings at *m*_neu_ = 5, which is the neutral block length at which the incidence of defects is highest (Figure 5a). Analogous plots for different values of *m*_neu_ are provided in Figure S15.

The traces in Figure 9a present numerous switches between positive and negative values of , corresponding to inversions, or ″flips″, of the chemical orientation vectors. There are two key aspects of these stochastic inversions. First, the intervals between consecutive flips of a given ring vary widely, with an average duration of approximately 70× 10^2^τlj. This time scale is of the same order as the ring rotational time, , discussed previously. Second, the flips often occur in a coordinated manner across concatenated rings. In fact, the traces show rapid sequences of flips involving multiple consecutive rings.

Figure 9b illustrates the elementary steps of the propagating chemical orientation inversions. The snapshots correspond to the two time points, *t*_1_ and *t*_2_, marked by vertical dashed lines in panel (a). The snapshots show the hopping of a defect between neighboring rings. At *t*_1_, the defect is located at the contact region of the two leftmost rings, *i* = 5 and 6, which interface through their neutral blocks. At *t*_2_, the defect has hopped to the right, i.e., to the interface of rings *i* = 6 and 7. This defect migration is driven by a half-turn rotation of ring 6, which flips the charged and neutral blocks in contact with its neighboring rings.

To summarize, the results of Figure 9 show that at *m*_neu_ = 5, chemical reorientations of individual rings occur abruptly, with time separations of the order of , although with significant variance. These reorientations are typically part of a triggered cascade of defect hops that propagate across neighboring rings.

## Conclusions

In this study, we considered model polycatenanes made of diblock copolyelectrolyte (co-PE) rings in solution with counterions. Using Langevin dynamics simulations, we investigate how the static and dynamic properties depend on ring composition. The latter was changed by systematically varying the number of monomers of the neutral block, *m*_neu_, while keeping fixed the number of ring monomers (*m* = 20) and of linearly concatenated rings (*n* = 12).

Our results revealed an unexpectedly complex dependence on ring composition of several metric and dynamical observables. Specifically, the catenane’s radius of gyration, the mechanical bond length, the chemical orientation of consecutive rings, and the rotational relaxation times of individual rings were found to be nonmonotonic with *m*_neu_, all presenting a maximum for *m*_neu_ ≈ 5. These nonmonotonicities arise from the competition between the intra- and inter-ring electrostatic repulsion, which tends to pull rings away, and mechanical bonding, which maintains neighboring rings in spatial proximity.

This tug-of-war takes on different forms depending on the balance of charged and neutral block lengths, leading to qualitatively different properties. For *m*_neu_ < 5, the repulsion of the like-charged monomers is sufficiently strong to keep rings swollen and prevent their contact. At *m*_neu_ = 5, the neutral block length is large enough that neighboring rings can touch each other at their neutral-charged (or neutral–neutral) interfaces. At the same time, the inter-ring repulsion induces a tightening of the mechanical bonds. Because the neutral blocks co-opted in the succession of neutral-charged interlockings are short, consecutive rings are significantly restricted in their relative positioning, with two main consequences. On the one hand, consecutive chemical orientation vectors, defined as the distance vectors between the charged and neutral blocks midpoints, are strongly aligned. On the other hand, the local relaxation dynamics is hindered.

Increasing the neutral block length, *m*_neu_ > 5, lessens these conformational constraints, and so does reducing *m*_neu_ too, because going toward the fully charged limit eliminates the tight contacts of consecutive rings. It is for these reasons that the aforementioned metric and dynamic observables all present a maximum at *m*_neu_ = 5.

Finally, for *m*_neu_ > 10, where the majority of the monomers are neutral, the neutral blocks co-opted in the mechanically bonded regions are large enough, and the inter-ring repulsion is sufficiently small that consecutive rings are largely unrestricted, both orientationally and positionally, to the point that the neutral block of one ring is frequently interfaced with the charged blocks of both its neighbors. In such conditions, the variations of the metric observables, such as the ring’s and catenane’s size, are mainly ascribable to the progressive crumpling of the rings as the number of same-charged monomers is reduced.

Overall, two co-PE compositions, *m*_neu_ = 5 and 10, emerge as particularly noteworthy, as various metric and dynamical observables exhibit extremal behavior at these values. The case *m*_neu_ = 5 corresponds to the minimal neutral-block length needed to sufficiently reduce electrostatic repulsion so that rings can make contact at neutral-charged interfaces. Instead, *m*_neu_ = 10 corresponds to the shortest neutral blocks that contact the charged segments of both neighboring rings. We expect that this physical rationale for singling out relevant co-PE compositions would remain valid when the length and number of concatenated rings are changed, although these specific numerical values of *m*_neu_ would likely shift.

The findings open several avenues for further research. A natural extension would be varying the number of rings and monomers per ring, which are likely to influence the catenane’s flexibility, change the interplay of the local and global relaxation dynamics, and affect the number of defects and their interactions, too. Introducing rings with multiple alternating charged and neutral blocks could be a further design element for controlling the catenanes’ structural organization and dynamics. It would also be relevant to investigate how facile externally tunable conditions, such as solvent quality, pH,^60^ and concentration and nature of counterions in solution,^35−37,41−43,58^ could modulate the properties of co-PE catenanes across different scales, providing insights that could be transferrable to more general and complex classes of mechanically bonded supramolecular constructs. Furthermore, given that our dynamic characterization relied on conventional Langevin simulations, it would be worthwhile to assess in future studies whether significant differences arise when hydrodynamic effects are included.

As for the impact that varying solution composition may have, one may, *de facto*, attempt an educated guess on how the highlighted properties could be impacted basing on our previous results on knotted copolyelectrolytes and the pair distribution functions shown in Figure S9. Thus, adding monovalent salts is likely to “crumple” rings^35,58^ reducing their gyration radius, the mechanical bond lengths, and the inter-ring repulsion; in turn, this is likely to foster the decrease in the overall system’s gyration radius and “end to end” distance. As for the relative orientation of subsequent rings, the propensity of being positively correlated is expected to decrease, albeit it should not be vanquished as 1:1 salts do not appear able to completely neutralize charged segments due to condensation. The impact of divalent counterions or salts with divalent species with opposite charge than chains’ ones should, instead, be more marked (see ref (35) for knotted species) with respect to “ring crumpling” and all related metrics. Conversely, it appears difficult to predict the effect on the orientation of copolymer rings, as divalent species may effectively bridge charged segments, thus limiting the impact of neutral ones. Investigating the latter aspects may be of general interest, even though it is likely to be fraught with technical difficulties due to the possible increase in intra and inter-ring friction caused by the stronger electrostatic interaction between mobile ions and chains.

With respect to possible applications of the results discussed, one may envision the transfer of defects from one catenane end to the opposite one triggered by chemical-related or electrostatic stimuli. The system would, thus, act as a mechanically connected molecular wire (or switch^61^) rather than a covalently bonded one, as the defect migration could be interpreted in terms of an overall charge transfer. Moreover, one may harness the intrinsically polar nature of copolyelectrolyte catenanes in applications similar to the ones of covalently bound electrets^62^ when the macromolecular size range could be useful, for instance, as a separation layer or for its reorientation capability. Finally, the ability to control the number of defects may be exploited to trigger/impair energy or electron transfer along the catenane when the different chemical nature of the comonomers bestow them with the capability of acting as, alternatively, donors and acceptors.^63,64^

## Supporting Information Available

Additional results for the metric and dynamical properties of the considered block copolyelectrolyte catenanes (PDF)
