# Supplementary material for: Effect of Ring Composition on the Statics and Dynamics of Block Copolyelectrolyte Catenanes
Source: Macromolecules. 2025 Apr 18;58(9):4447–58. doi: 10.1021/acs.macromol.5c00099 (PMC12080470; doi:10.1021/acs.macromol.5c00099)
Supplement: Supplementary file 1 — ma5c00099_si_001.pdf [file ma5c00099_si_001.pdf]

Supporting Information
"Effect of ring composition on the statics and dynamics of block copolyelectrolyte catenanes"

by P. Chiarantoni, A. Tagliabue, M. Mella and C. Micheletti

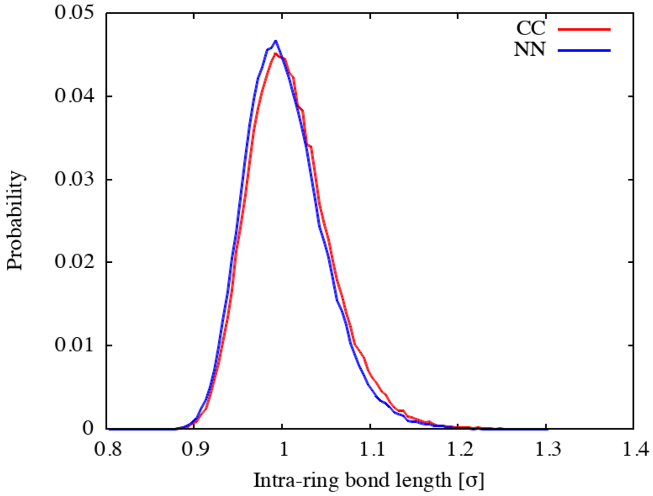

FIG. S1. Probability distributions of intra-ring bond lengths, i.e., the distance of consecutive monomers in a ring. The two curves correspond to consecutive monomers that are both charged (CC) or neutral (NN). The data, which was collected for the $m_{\rm neu} = 10$ case, show that the intra-ring bond distance does not vary significantly with the charged/neutral type of the monomers.

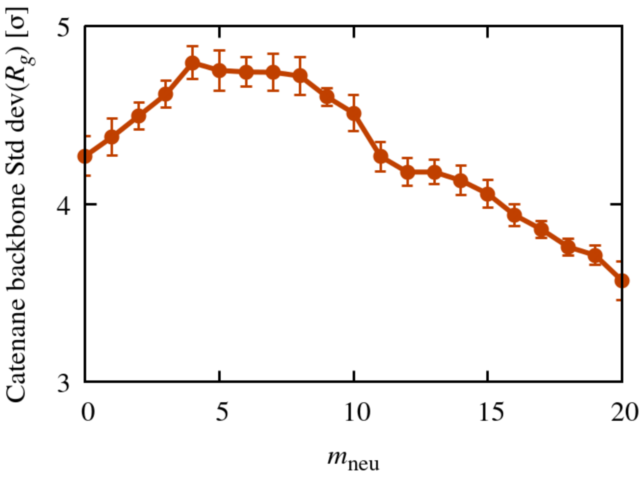

FIG. S2. Standard deviation of the gyration radius of the catenane backbone as a function of the ring composition, $m_{\rm neu}$.

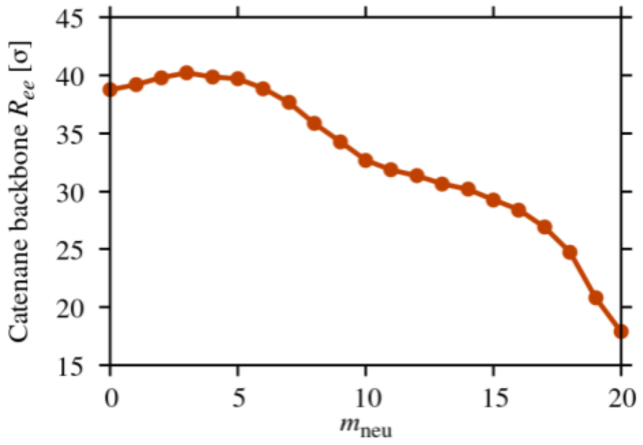

FIG. S3. End-to-end distance of the catenane backbone versus ring composition, m_{neu}.

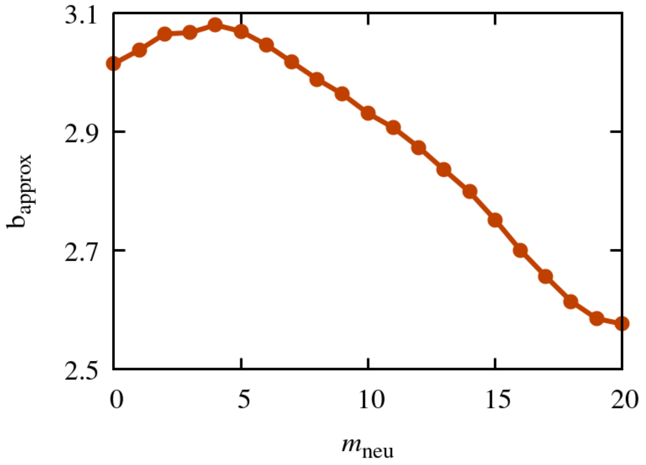

FIG. S4. Dependence on ring composition of the theoretical estimate of the mechanical bond length based on the idealized representation of the catenane as a one-dimensional series of elliptical rings of identical size and spacing, see main text. The mechanical bond length of such systems can be approximated as $b_{\text{approx}} = 2\,l_1 - d_{\text{min}}$, where l_1 is the square root of the principal eigenvalue of the gyration tensor and d_{min} is the distance of minimum approach of the interlocked rings. Substituting in the expression the observed values of l_1 and d_{min}, see Fig. S7 and Fig. 3, yields the shown curve, which features the same non-monotonicity of the observed mechanical bond length (Fig. 3).

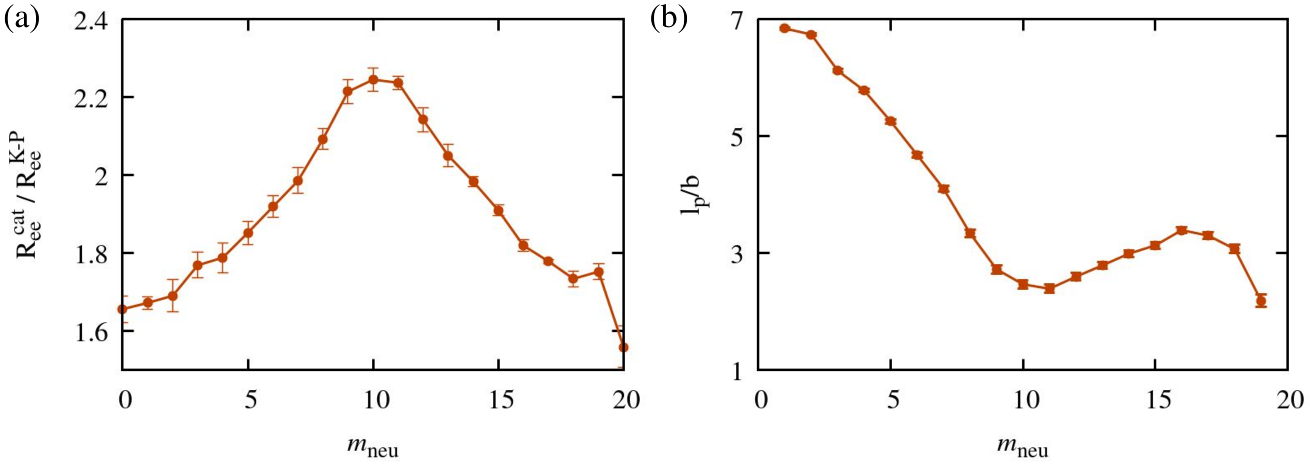

FIG. S5. (a) Ratio between the root mean squared end-to-end distance of the catenane backbone and the equivalent Kratky-Porod (KP) chain as a function of the ring composition, $m_{\rm neu}$. The mean squared end-to-end distance of the equivalent KP chain was computed as $R^2_{\rm ee,KP} = b^2 \sum_{i,j=1}^{n-1} \exp\left[-|i-j|/(lp/b)\right]$, where $n = 12$ is the number of rings in the catenane and b is the average mechanical bond length. The adimensional ratio l_p/b is the effective persistence length of the mechanical backbone computed from the orientational correlation of consecutive (normalized) mechanical bond vectors, $l_p/b = -(\ln\langle \hat{b}_i \cdot \hat{b}_{i+1}\rangle)^{-1}$. The dependence of l_p/b on the ring composition $m_{\rm neu}$ is shown in panel (b).

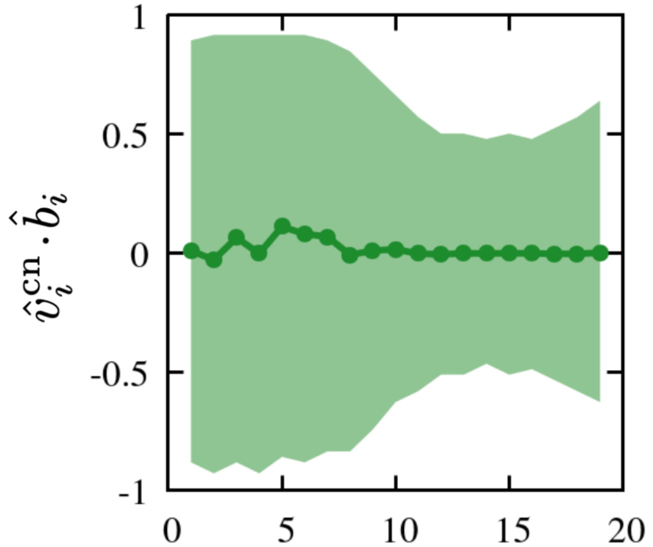

FIG. S6. Orientational correlation of the chemical and mechanical bond vectors for varying ring composition. The data points represent the average scalar products of the normalized chemical orientation vector of a ring, $\hat{v}_i^{\rm cn}$, and one of its two mechanical bond vectors, $\hat{b}_i$. The shaded band indicates the Q1-Q3 interquartile range, from the 25th (Q1) to the 75th (Q3) percentiles. The figure complements Fig. 4 of the main text, where the modulus value, $|\hat{v}_i^{\rm cn} \cdot \hat{b}_i|$, was considered to discount the $\hat{b}_i \to -\hat{b}_i$ symmetry, due to the equivalence of direct and inverted backbone orientations. The equivalence is reflected by the mirror symmetry of the plot with respect to the zero value.

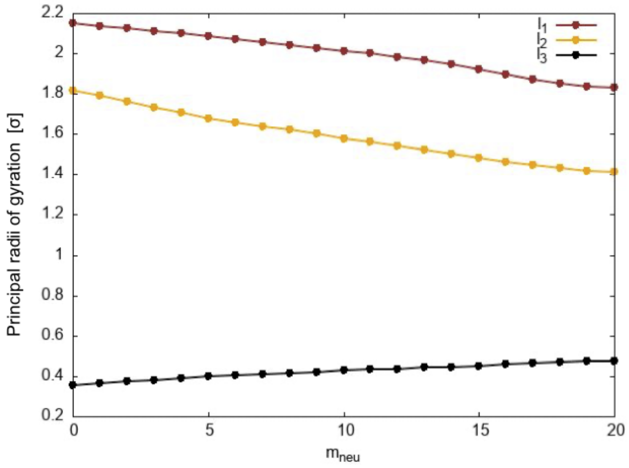

FIG. S7. Square roots of the gyration tensor eigenvalues (i.e. principal gyration radii) for individual concatenated rings, as a function of ring composition. At each value of $m_{\rm neu}$, the two largest radii, l_1 and l_2, are comparable and much larger than the third one, l_3, indicating that concatenated rings are approximately circular and planar. The approximate planar circular state is better realized as the system approaches the fully charged ring case due to the intra-ring electrostatic repulsion.

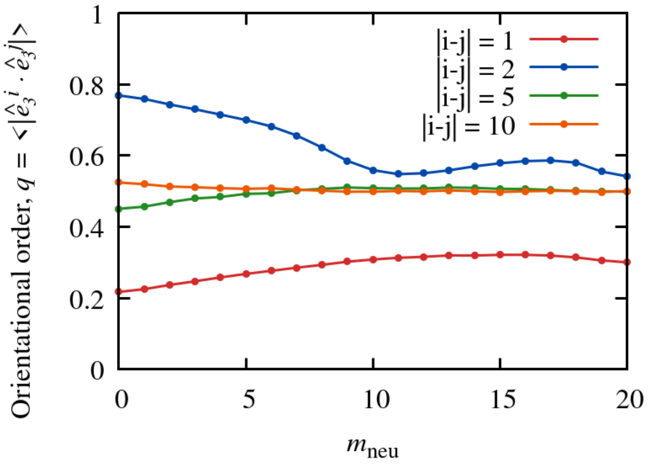

FIG. S8. Planar orientational order of rings at different sequence separations and compositions. The planar orientational order of rings i and j was measured with the absolute value of the scalar product of the eigenvectors of the gyration tensor associated with the smallest eigenvalues, $\langle|\hat{e}_3^i \cdot \hat{e}_3^j|\rangle$, where $\langle\rangle$ denotes averaging over the sampled conformations. The eigenvectors correspond to the normal of the rings' osculating planes.

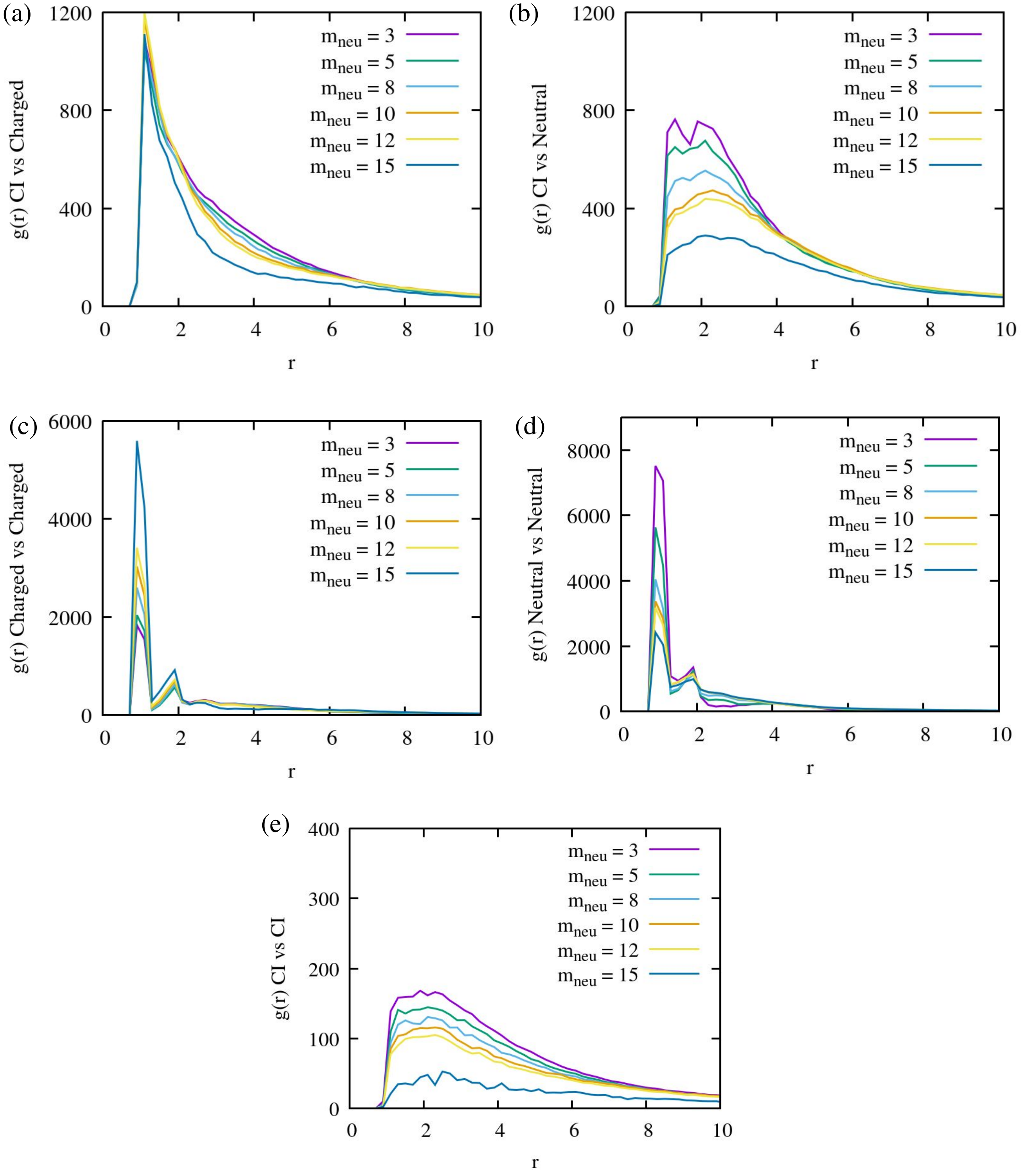

FIG. S9. Radial distribution functions for all different pairs of species in the system: counterions (CI), charged, and neutral monomers. The radial distribution functions are presented for various values of the ring composition, $m_{\rm neu}$. The large $g(r)$ values reflect the proximity induced by the chain connectivity and concatenation of the rings.

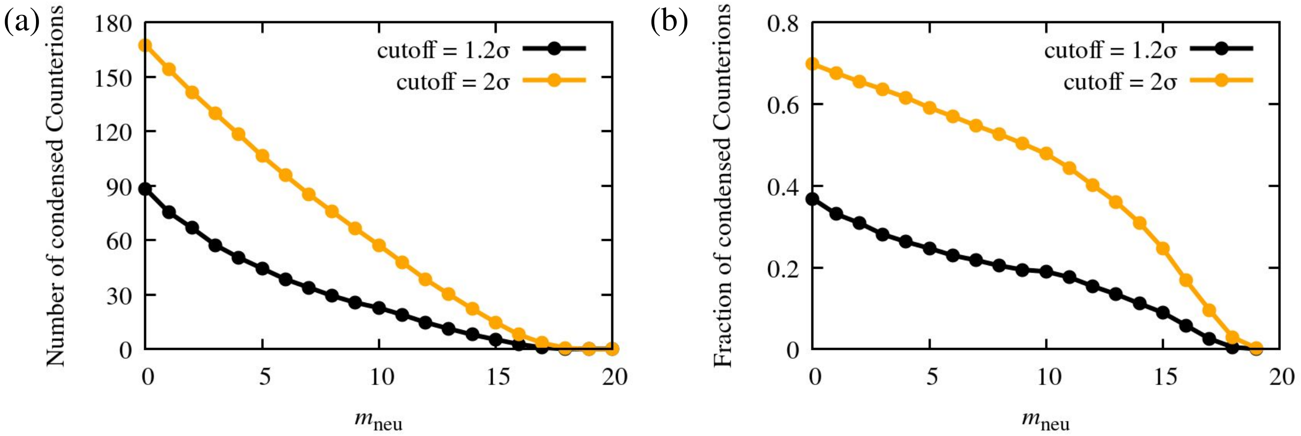

FIG. S10. (a) Number and (b) fraction of counterions condensed on the catenane as a function of the ring composition, m_{neu}. We considered two cutoff distances: (i) 2σ corresponding to the system's Bjerrum length and (ii) 1.2σ, corresponding to the peak of the radial distribution function of counterions and charged monomers, see Fig. S9.

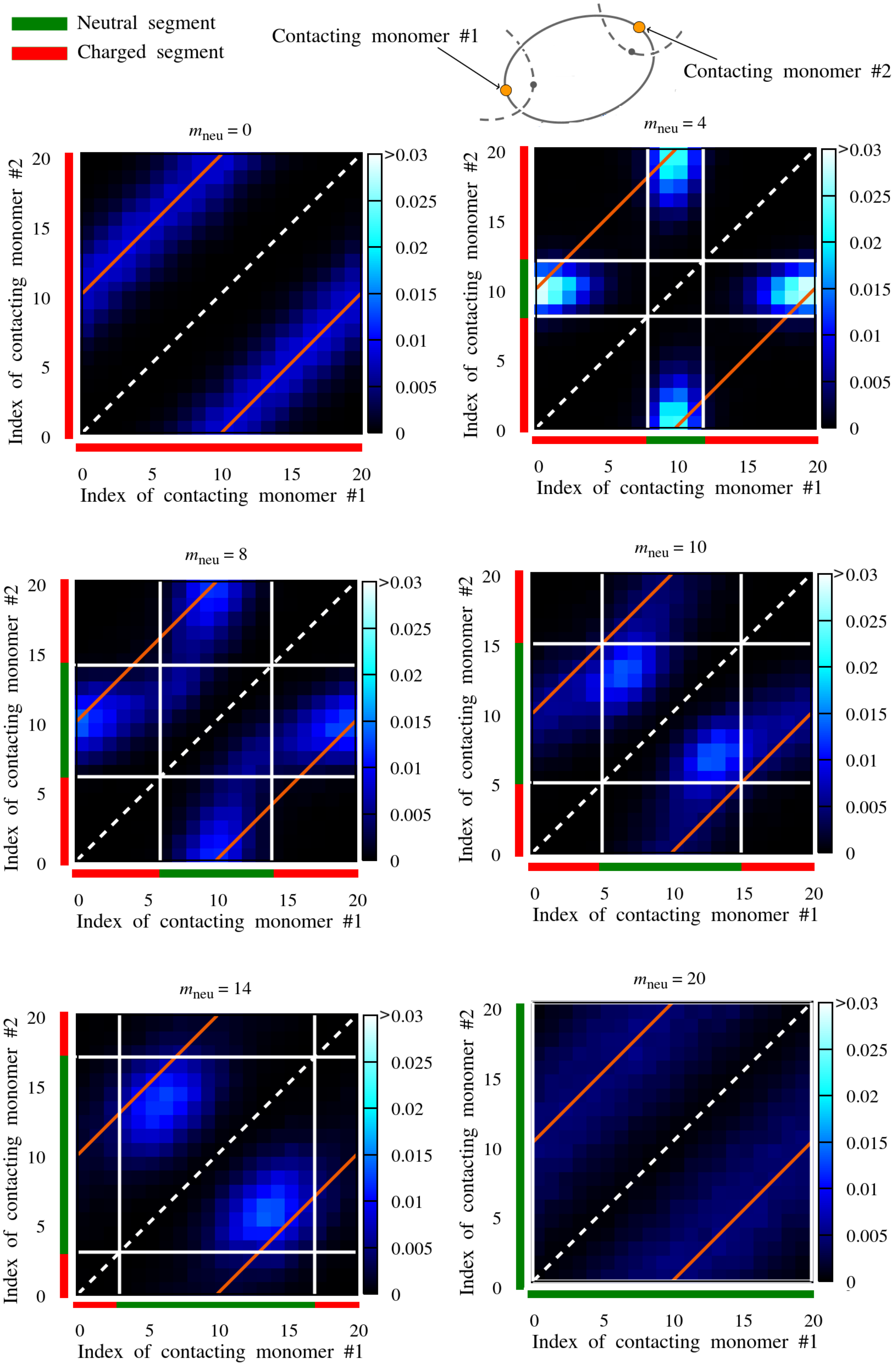

FIG. S11. Probability distribution of mechanically-bonded monomers in a given ring. The heatmaps represent the joint probability distribution of the indices of the two monomers of the central ring in the catenane closest to each neighboring ring, as sketched on the top. The heatmaps are shown for several different values of m_{neu}, with the neutral (green) and charged (red) character of the monomers indicated by the colored sidebars. The diagonal symmetry of the heatmaps, reflecting the equivalence of the two monomers, was not imposed on the data.

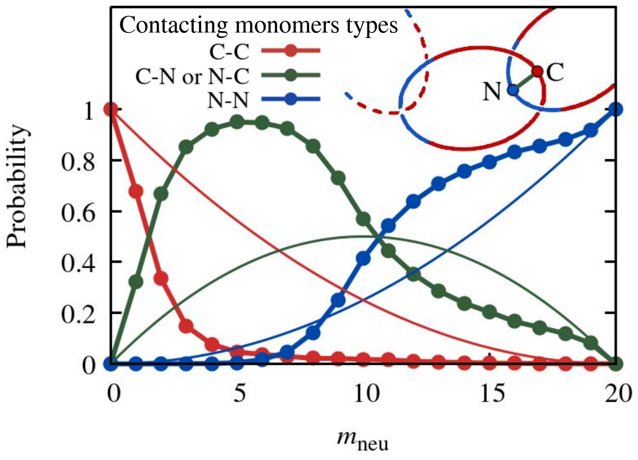

FIG. S12. Contact probability of neutral and charged monomers as a function of ring composition. The curves show the $m_{\rm neu}$ dependence of the probability that the two closest monomers of concatenated rings are both neutral (N-N), both charged (C-C) and of different types (C-N or N-C). The probabilities are normalized at each $m_{\rm neu}$ value. The three thin colored curves represent mean-field-like approximations; see the main text. The data complement those of Fig. 7 of the main text in that the contact probabilities shown here refer to a single mechanically-bonded interface in the middle of the catenane. In contrast, the data in Fig. 7 are the number of contacts summed over the $n - 1 = 11$ interfaces.

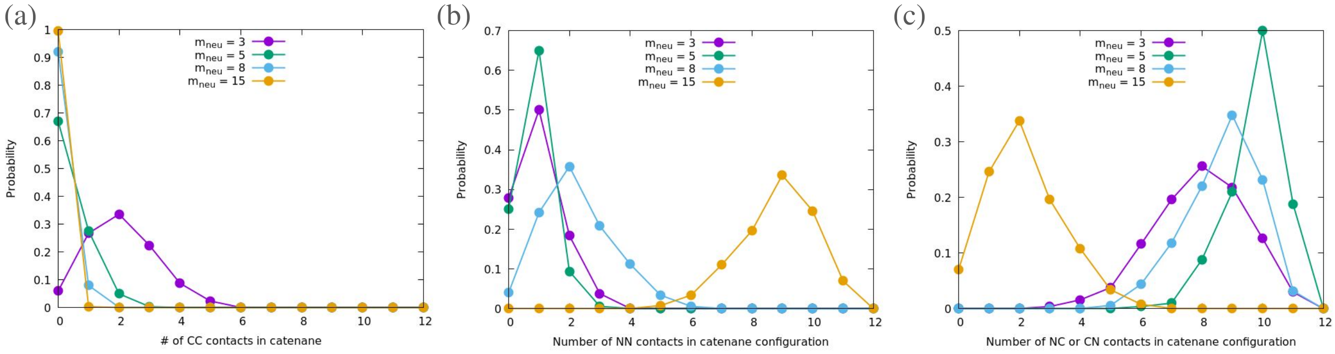

FIG. S13. Probability distributions of the number of C-C, N-N, and N-C/C-N contacts in a catenane at different ring compositions. The data pertain to the number of neutral-neutral (N-N), charged-charged (C-C), and mixed (C-N or N-C) pairs of contacting monomers in the catenane, and the contacts refer to the two closest monomers of concatenated rings.

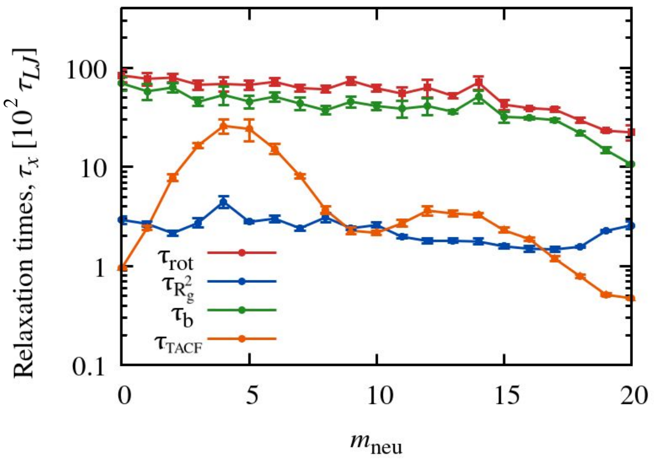

FIG. S14. Characteristic relaxation times of global and local metric observables. The curves show the $m_{\rm neu}$ dependence of the characteristic rotational times of the entire catenane, $\tau_{\rm rot}$, and of a single mechanical bond vector, $\tau_{\rm b}$. $\tau_{R_g^2}$ is the correlation time of the mean square gyration radius of the catenane, and $\tau_{\rm TACF}$ is the characteristic decay time of the orientational correlation function of the ring's diameters, averaged over all diameters.

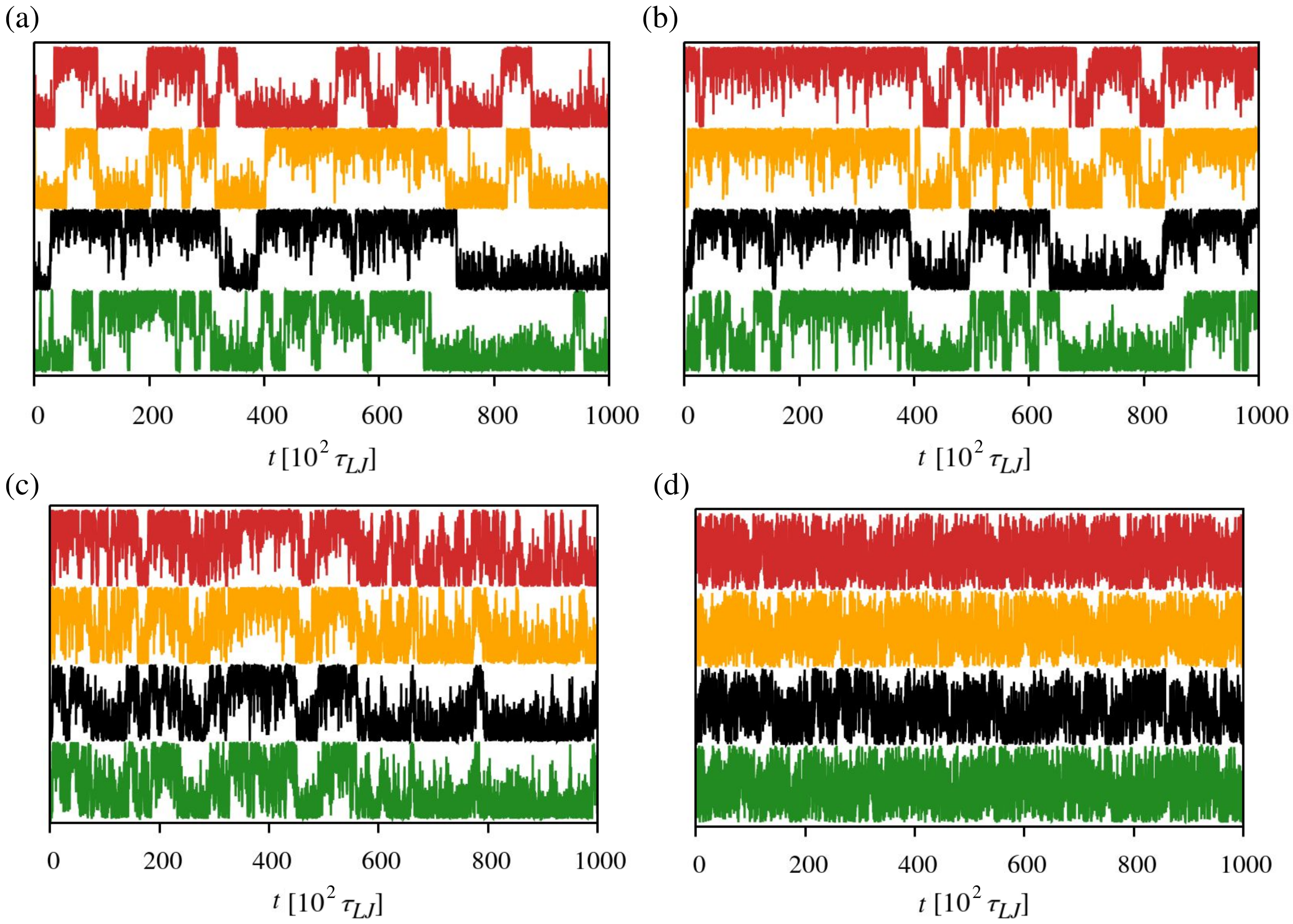

FIG. S15. Dynamics of chemical orientation defects. The traces represent the time evolution of the orientational correlation (scalar product) of consecutive charged-to-neutral vectors, $\hat{v}_i^{\rm cn}$ and one of the two corresponding mechanical bonds $\hat{b}_i$. The plots are analogous to those of Fig. 9a of the main text. The data are for a stretch of a few consecutive rings, $i = 5, 6, 7, 8$ at various ring compositions, $m_{\rm neu} = 3, 5, 8, 15$ in order from panel (a) to (d)

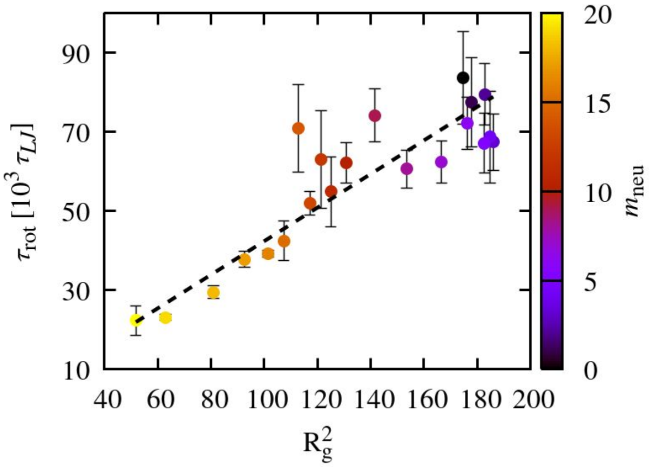

FIG. S16. Scatter plot of the characteristic re-orientational time of the catenane backbone, $\tau_{\rm rot}$ versus the mean square gyration radius of the backbone. Data points for different ring compositions are color-coded according to the bar on the right. The dashed line is a fit of the data based on a proportionality relationship. Neglecting counterion effects on diffusivity, the approximate proportionality suggests a Rouse-like relaxation for the catenane backbone.
